# Accuracy Evaluation of LLM-Generated Electronic Health Record Interpretations Against Medically Verified Sources

**DOI:** 10.3390/bioengineering13101123

**Published:** 2026-09-26

**Authors:** Mia Rovis, Alma Smajić, Marijela Miličević, Sandi Baressi Šegota, Vedran Mrzljak, Antun Gršković, Juraj Ahel, Klara Smolić, Dean Markić, Ivan Lorencin

**Affiliations:** 1 Faculty of Informatics, Juraj Dobrila University of Pula, Alda Negrija 6, 52100 Pula, Croatiaalma.smajic@unipu.hr (A.S.); marijela.milicevic@unipu.hr (M.M.); sandi.baressi.segota@unipu.hr (S.B.Š.); ivan.lorencin@unipu.hr (I.L.); 2 Faculty of Engineering, University of Rijeka, Vukovarska 58, 51000 Rijeka, Croatia; 3 Faculty of Medicine, University of Rijeka, Brace Branchetta 20, 51000 Rijeka, Croatia; antun.grskovic@medri.uniri.hr (A.G.); juraj.ahel@medri.uniri.hr (J.A.); klara.smolic@uniri.hr (K.S.); dean.markic@medri.uniri.hr (D.M.); 4 Department of Urology, Clinical Hospital Center Rijeka, Krešimirova 42, 51000 Rijeka, Croatia

**Keywords:** large language models, urology, medical findings, interpretation, clinical accuracy

## Abstract

This study evaluates the accuracy of medical-report interpretations generated by large language models in comparison to medically verified sources, with a particular focus on urology. The main goal is to examine the extent to which LLMs can reliably and precisely explain medical findings, with emphasis on expert urological terminology. For the evaluation of interpretation accuracy, real clinical documentation was used, specifically a dataset of 40 urological reports from the Clinical Hospital Center Rijeka. The generated explanations were compared with medically correct definitions using quantitative metrics assessing interpretability, specificity, and clinical accuracy. The results revealed a clear stratification of model performance, with DeepSeek-V3.2, GLM-4.6, MiMo-V2-Flash, and Qwen2.5-7B-Instruct achieving the highest semantic accuracy and consistency, while several models exhibited unstable behavior characterized by occasional catastrophic failures. Overall, the findings indicate that although LLMs can produce clinically coherent explanations of urological findings, their variability and susceptibility to hallucinations necessitate human oversight, supporting their use primarily as decision-support tools rather than autonomous clinical interpreters.

## 1. Introduction

The rapid digitalization of healthcare has led to a steady increase in electronically stored clinical documentation, creating new opportunities, alongside new risks, for automated interpretation of medical findings in routine care. In parallel, the integration of LLMs into clinical workflows has demonstrated significant potential for enhancing healthcare delivery, yet their application in interpreting specialized medical findings remains insufficiently validated across different medical domains. Moreover, their deployment introduces substantial challenges related to factual accuracy, interpretability, model bias, ethical considerations, data privacy, and regulatory compliance [1].

Recent healthcare-specific literature has further emphasized the importance of patient privacy, data governance, fairness, transparency, accountability, human oversight, and regulatory considerations when deploying AI systems in clinical environments [2,3]. These dimensions are particularly critical in specialized domains such as urology, where misinterpretation of findings can have direct clinical consequences.

On this note, while electronic health records (EHRs) contain vast amounts of structured and unstructured clinical data [4], the interpretation of domain-specific medical terminology, particularly in specialized fields such as urology, presents substantial challenges for both patients and non-specialist healthcare providers. Recent work has shown that models like LLaMA 3 [5] and Mistral 7B [6] demonstrate strong general language capabilities, but their performance in specialized medical domains requires systematic evaluation. For urology specifically, studies have begun assessing LLM applications [7,8,9], yet questions remain about their reliability for interpreting authentic clinical documentation.

Although LLMs can generate fluent explanations [10], their outputs can include omissions, clinical misinterpretations or hallucinated statements, which can be particularly harmful when users rely on them to understand abnormal findings. Research on hallucination mitigation [11,12] has identified various sources of these errors, highlighting the need for robust evaluation frameworks. Therefore, beyond general language quality, clinical deployment requires rigorous evaluation frameworks that assess not only semantic similarity but also clinical correctness, specificity, and alignment with authoritative medical literature [13].

What is more, despite growing research on LLM applications in clinical decision support and EHR summarization, there is limited empirical evidence evaluating the accuracy of LLM-generated interpretations of urological findings against medically verified sources. This leaves a critical gap in understanding their reliability for specialized medical domains [14]. Interpretation focused on urology tasks differs from general clinical Q&A. They require faithful explanation of expert terminology embedded in real documentation, where even small inaccuracies can distort meaning [15]. Accordingly, evaluation should reflect the real user setting in which a patient asks about uncertainty in their own report and expects an explanation that is complete, relevant, and concise, without introducing unsupported claims. As noted in recent work on trustworthy AI in healthcare [16], such evaluations must prioritize clinical safety alongside technical performance.

The primary objective of this study is therefore to evaluate the clinical correctness of LLM-generated interpretations of urological findings against medically verified reference definitions. Secondary objectives include comparing the performance of multiple LLMs under identical conditions and assessing the contribution of retrieval-augmented generation (RAG) to interpretation quality.

This study addresses the gap by systematically evaluating how accurately LLMs interpret urological findings from University Hospital Centre Rijeka, using medically verified definitions as the reference standard. Interpretation accuracy was measured using real clinical documentation, consisting of a dataset of 40 urological reports. Explanations generated by nine large language models were compared with medically verified definitions drawn from relevant medical literature and clinical guidelines. To enhance reliability and reduce unsupported content, a custom retrieval-augmented generation (RAG) workflow was implemented, building on approaches like those described in [17]. This workflow retrieves and incorporates curated reference material relevant to each clinical finding before the model produces its interpretation, ensuring that the generated explanations are grounded in verified medical knowledge and directly comparable to the reference standards.

The novelty of this study lies in three key aspects: (1) the use of authentic EHR-derived urological findings rather than synthetic or textbook cases; (2) a multi-model comparison involving eight LLMs evaluated under identical conditions; and (3) the integration of a retrieval-augmented generation (RAG) framework grounded in medically verified sources. These differentiators distinguish this work from previous LLM evaluations in urology, which have primarily relied on synthetic cases or single-model assessments.

In summary, this combined evaluation design provides the distinction between interpretations that are merely linguistically plausible and those that are clinically accurate, closely aligned and appropriately concise for patient-facing communication. By emphasizing specialized urological terminology within real EHR documentation and grounding the assessment in medically verified sources, the study provides domain-specific evidence on the reliability and practical applicability of explanation tools, highlighting key considerations for their safe implementation in medical workflows.

The remainder of this paper is structured as follows. Section 2 reviews existing research on the use of LLMs in clinical environments, with particular emphasis on clinical decision support, evaluation benchmarks, and identified limitations such as reliability, bias, and guideline adherence, and formulates the research hypotheses addressed in this study. Next, in Section 3, the dataset of anonymized urological EHR reports is described alongside the preprocessing and segmentation procedures, the structured evaluation framework including retrieval-augmented generation, the evaluated language models, the automated semantic evaluation metrics and statistical analysis used for performance assessment. Subsequently, Section 4 presents the quantitative evaluation of model performance, including aggregate statistics, comparative rankings, semantic similarity analysis, and qualitative examination of typical error patterns and representative cases. This is followed by Section 5, which interprets these findings in the context of existing literature, highlighting both the strengths and limitations of LLM-generated clinical interpretations, as well as their implications for clinical use, study limitations, and directions for future research. Finally, Section 6 summarizes the main findings of the study, emphasizing the potential of LLMs as supportive tools for interpreting specialized medical terminology while underscoring the need for clinically grounded evaluation and cautious deployment in real-world healthcare settings.

## 2. Background and Related Work

Recent research on LLMs in clinical environments has expanded rapidly, with the literature largely converging around two principal domains: clinical decision support and patient safety, alongside automation of clinical documentation through EHR summarization. Across these domains, studies consistently report meaningful potential for improving workflow efficiency and enabling human, AI collaboration, while simultaneously identifying persistent limitations in clinical reasoning reliability, diagnostic interpretation, and adherence to evidence-based guidelines. Collectively, these findings underscore the necessity for rigorous validation prior to real-world clinical deployment, particularly in safety-critical medical contexts [18].

Benchmarks play a crucial role in this validation process. The GLUE benchmark [19] has become a standard for evaluating general natural language understanding, while domain-specific benchmarks like ClinicBench [20] and medical agent frameworks [21] are emerging for clinical applications. Evaluation metrics themselves have also evolved, with approaches like BERTScore [22] providing more nuanced semantic similarity assessment than traditional lexical metrics. Studies on medical question answering [23] have demonstrated both the potential and limitations of LLMs in clinical contexts.

Within clinical decision support specifically, converging empirical evidence indicates that current LLMs remain unsuitable for autonomous clinical decision-making. Structured evaluations using real clinical datasets demonstrate recurrent failures in guideline adherence, diagnostic reasoning, and laboratory interpretation, with degradation observed not only in final diagnostic outputs but also across intermediate reasoning processes. These observations highlight the importance of process-level evaluation frameworks rather than reliance on outcome-only accuracy metrics [18]. Furthermore, randomized clinical evidence suggests that access to LLM assistance does not inherently improve physicians’ diagnostic reasoning, despite strong standalone model performance. This discrepancy indicates that clinical benefit depends on effective workflow integration, interpretability, and calibrated trust rather than model capability alone [24]. At the same time, comparative diagnostic studies report that GPT-4-class models can achieve high diagnostic accuracy on structured clinical case presentations and, in controlled experimental settings, performance comparable to or exceeding that of trainee physicians. However, such findings remain strongly dependent on study design and evaluation context and therefore do not guarantee safe real-world clinical deployment, reinforcing the critical distinction between benchmark performance and clinical reliability [25,26]. Chain-of-thought prompting [27] has shown promise for improving reasoning in some contexts, but its effectiveness in clinical settings remains variable.

On this note, comparative analyses between generative LLMs and specialized diagnostic decision-support systems further indicate that domain-specific AI solutions may achieve superior diagnostic correctness, suggesting that general-purpose language models currently lack the specialization required for consistently reliable clinical reasoning [28]. In parallel, systematic investigations identify the presence of bias across clinical LLM applications, with potential implications for diagnostic error and inequitable care, thereby reinforcing the need for robust evaluation, continuous monitoring, and domain-specific validation prior to clinical integration [29].

However, the extent to which these limitations translate into specialized clinical domains remains insufficiently understood. Despite the growing evidence base addressing general clinical reasoning and decision support, specialized medical domains remain comparatively underexplored. Interpretation of domain-specific findings embedded within authentic EHR documentation requires high terminological precision, contextual fidelity, and strict alignment with authoritative medical knowledge, where even minor inaccuracies may alter clinical meaning or patient understanding. Urology-focused evaluations indicate that, although LLMs can generate coherent and educationally useful responses, their medical accuracy and safety remain inconsistent, with specialist clinicians consistently outperforming LLMs in the correctness and reliability of urological guidance [30]. Recent studies have specifically examined ChatGPT’s performance in urology [8] and the quality of patient education materials generated by LLMs [9], with mixed results. A comprehensive review of LLM applications in urology [7] highlights both opportunities and challenges for the field. These findings reinforce the principle that linguistic fluency does not equate to clinically trustworthy interpretation, particularly in patient-facing contexts involving abnormal findings and diagnostic uncertainty.

Within the broader literature, including several studies published in *Expert Systems with Applications* (ESWA), our work aligns with a growing body of research on applying large language models to clinical text processing and medical decision support. Several recent ESWA studies have established foundational approaches that our work extends into, within the specialized domain of urological interpretation. Li et al. [31] evaluated LLMs on semantic similarity tasks in acupuncture diagnosis, establishing a paradigm of using BERTScore for medical interpretation assessment, a methodology we adopt and extend by applying it to urological EHR data within a RAG framework. Similarly, the clinical information extraction pipeline developed by Wiest et al. [32] using locally deployed Llama 2 for extracting clinical features from free text demonstrates the feasibility of using open-source LLMs to process real clinical documentation, a principle we extend from feature extraction to full clinical interpretation. In this vein, the two-stage proactive dialogue generator proposed by Li et al. [33] for efficient clinical information collection further underscores the importance of structured, multi-step LLM workflows in healthcare settings. Additionally, LEGOLAS, the explainable predictive process monitoring method developed by Pasquadibisceglie et al. [34], uses fine-tuned LLMs for early and explainable prediction of hospital admissions from emergency department patient journeys. However, while these studies address various aspects of clinical text processing, clinical dialogue, predictive monitoring and diagnostic evaluation, none of them specifically evaluate the accuracy and hallucination risks of LLM-generated interpretations of specialized urological findings against medically verified reference standards within a RAG framework. Our study fills this gap by providing a quantitative, multi-model evaluation of LLM-generated urological interpretations using real-world EHR data, systematically assessing both lexical precision and semantic alignment. Furthermore, it explicitly identifies catastrophic failure patterns that render certain models clinically unsuitable. In doing so, we extend the existing ESWA discourse from general clinical NLP and LLM evaluation into the critical domain of specialist terminology interpretation, offering novel evidence on model reliability, stability, and safety in urological contexts.

Taken together, the current literature demonstrates that while LLMs show promise in decision-support augmentation and structured diagnostic reasoning, substantial concerns persist regarding reliability, bias, guideline adherence, and domain specificity. From this, the following research hypotheses are derived:

1. LLM-generated interpretations may show inconsistencies with established clinical guidelines when evaluated against reference standards.
2. Different LLMs may vary substantially in correctness and reliability when interpreting specialized urological findings.
3. Linguistic fluency of LLM outputs does not guarantee clinically correct interpretation.

In other words, there is a lack of studies that have quantitatively evaluated the clinical correctness of LLM-generated interpretations of authentic urological EHR findings against authoritative medical reference definitions using structured accuracy metrics and retrieval-grounded generation. This study directly addresses this gap by providing a quantitative, medically grounded evaluation of LLM-generated interpretations of real urological findings based on validated reference definitions and clinically oriented accuracy measures. Establishing such evidence is essential for determining whether contemporary LLM systems can progress from experimental language tools toward clinically reliable components of medical information interpretation and for informing future regulatory, safety, and deployment frameworks in specialized healthcare domains.

## 3. Materials and Methods

This section describes the dataset, experimental framework, and evaluation methodology used to assess the performance of large language models in generating clinically accurate interpretations of urological findings.

### 3.1. Dataset and Data Preparation

The dataset used in this study consists of forty anonymized urological EHR reports collected from routine clinical practice. The reports contain descriptive clinical findings related to urological examinations and diagnostic procedures. Prior to analysis, all records were fully anonymized to remove patient identifiers and any potentially sensitive metadata, in accordance with ethical approval obtained from the Institutional Review Board of Clinical Hospital Center Rijeka (Approval No. KLASA: 003-05/19-1/26, UR.BROJ: 2170-29-02/1-19-2; 14 March 2019). Each report was then processed to extract clinically relevant textual segments describing findings or diagnostic observations. Following preprocessing, the extracted clinical findings were segmented into smaller textual units to facilitate controlled evaluation by the LLMs. Each segment served as an independent input instance for the experimental pipeline. This approach ensured that the models processed manageable text chunks while preserving the clinical context necessary for accurate interpretation.

The dataset comprises a diverse range of urological findings spanning multiple subdomains, including prostate-related findings (e.g., PSA levels, prostate volume, BPH), kidney findings (e.g., hydronephrosis, renal cysts, parenchymal thinning), bladder findings (e.g., bladder wall thickening, urinary retention), and other urological conditions (e.g., urinary tract infections, hematuria, stones). The extracted text segments ranged in length from 5 to 45 words, with an average length of approximately 18 words.

To address the question of diagnostic difficulty, findings were stratified by complexity level based on expert assessment. Of the 40 clinical findings, approximately 28 (70%) were classified as straightforward (e.g., common terms such as PSA, BPH, hydronephrosis), while 12 (30%) were classified as complex (e.g., rare findings, ambiguous terminology, or findings requiring integration of multiple clinical parameters). This stratification was used to analyze model performance across different difficulty levels.

The dataset consists of real, anonymized EHR reports and cannot be publicly shared due to patient privacy and ethical restrictions. The data are available from the corresponding author upon reasonable request and with appropriate institutional approval.

### 3.2. LLM Evaluation Framework

A structured evaluation framework was designed to ensure consistent comparison between the tested LLMs. The system pipeline consisted of several sequential components: preprocessing, text segmentation, retrieval augmentation, language model inference, and automated evaluation.

In the preprocessing stage, clinical text segments were cleaned and prepared for model input. These segments were then passed through a retrieval component that supplied contextual information relevant to the identified clinical term or finding. The retrieved context was combined with the original input to support more accurate semantic interpretation. Each of the processed segments was subsequently submitted to the selected LLM for definition generation.

In this study, the term “interpretation” is used as the overarching term for the overall task performed by the model (generating a clinically meaningful explanation of a urological finding), while “definition” refers specifically to the output format generated by the model (a concise medical definition of a term). This terminology is used consistently throughout the manuscript.

To further improve the quality and clinical relevance of generated definitions, the study implemented a RAG framework similar to that described in [17]. In this approach, external textual context was retrieved and incorporated into the model input before the generation step. For each clinical finding or urological term, relevant contextual information was retrieved from the available dataset and appended to the model prompt. This helped the LLMs in grounding their responses in clinically relevant material rather than relying solely on internal parametric knowledge.

It is important to clarify that the RAG component retrieves curated reference definitions and relevant clinical contextual information from medical literature and clinical guidelines, rather than information derived from the study dataset itself or directly from EHR texts. This ensures that generated interpretations are grounded in authoritative medical knowledge.

To add to this, the use of retrieval augmentation is particularly important in medical applications, where precise terminology and context-sensitive interpretation are required. In other words, by providing structured contextual input, the framework aimed to reduce hallucinations and improve the semantic consistency of generated definitions.

The prompt template was developed iteratively based on best practices from the literature on clinical LLM evaluation and refined through pilot testing to ensure that outputs were concise, definition-style, and clinically relevant. The final prompt template is presented in Table 1.

To ensure consistent responses across all evaluated models, a standardized prompting strategy was implemented. The prompt template instructed the model to generate a concise medical definition based exclusively on the provided text segment, along with several specified constraints that were designed to encourage focused, definition-style outputs rather than extended descriptive responses. Furthermore, the models were instructed to produce a clear and clinically accurate definition in English without including additional explanations, lists, or bullet points. This way, the medical terminology present in the input text was preserved and not translated. In addition, a system-level instruction defined the model’s role as a medical expert specialized in urology. This instruction encouraged the generation of clinically verified and precise definitions aligned with professional medical language and terminology.

Multiple models were evaluated to assess their ability to generate clinically accurate definitions of urological findings. Each model received identical input segments, retrieval context, and prompt instructions. For every clinical text segment, the models generated a single definition describing the identified term or finding. The outputs were stored in a structured format for subsequent evaluation and comparison, which ensured that each model processed the same dataset under identical conditions, allowing direct comparison of output quality. On this note, 8 LLMs were selected for evaluation based on their availability, ability to process medical text, and sufficient context window to handle individual extracted findings. Models that did not satisfy these minimum requirements were excluded from the evaluation to ensure fairness and comparability. The evaluated models included:

1. Mistral-7B-Instruct-v0.2 [6]
2. Qwen2.5-7B-Instruct [35]
3. gpt-oss-20b
4. GLM-4.6 [36]
5. DeepSeek-V3.2 [37]
6. Kimi-K2-Thinking [38]
7. MiMo-V2-Flash [39]
8. NVIDIA-Nemotron-Nano-9B-v2 [40]

For each model, interpretations were generated using a standardized prompt template, which was applied identically across all systems. The prompt instructed the model to produce a clear, concise medical definition of a given urological term without adding any information beyond the definition, which ensured that responses remained brief, focused, and comparable across models. Furthermore, the output format for each interpretation included a term definition as an explanation of the clinical finding in medically accurate terms and clinical context including any additional details necessary in the context of the term within the patient’s clinical scenario. All outputs were captured in a structured dataset linking each extracted finding to the corresponding model-generated interpretation, retrieved references, ground truth definitions and input segments. Moreover, no post-processing, prompt tuning, or model-specific optimization was applied, ensuring that evaluations reflect the raw interpretive capabilities of the LLMs. All in all, this structure supported reproducibility, fairness, and direct comparison across models while enabling downstream automated semantic and token-level evaluation.

### 3.3. Expert Validation

Two board-certified urologists (A.G. and J.A.) and one urology resident (K.S.) from the Clinical Hospital Center Rijeka participated in the study. Their involvement included: (1) verification of the reference definitions used as ground truth, ensuring that they accurately reflect established clinical knowledge; and (2) qualitative assessment of model outputs for the case studies presented in the Results section, including identification of clinically significant errors and hallucinations. The expert panel reviewed all 40 clinical findings and provided consensus-based reference definitions.

### 3.4. Automated Semantic Evaluation Metrics

To quantitatively evaluate the generated definitions, several automated semantic similarity metrics were applied. These metrics measured how closely the generated definitions correspond to the reference definitions extracted from the clinical dataset.

The primary metric used in this study was BERTScore [22], which evaluates semantic similarity using contextual embeddings derived from transformer-based language models. BERTScore calculates precision, recall, and F1-score by comparing token embeddings between generated and reference texts. Alongside BERTScore, cosine similarity was calculated between sentence embeddings representing the generated and reference definitions. This provided an additional measure of semantic alignment at the sentence level.

In addition, to obtain a comprehensive evaluation, multiple metrics were combined to assess both lexical overlap and deeper semantic similarity between the generated outputs and the reference definitions. Table 2 summarizes the evaluation metrics used in this study, including the level at which each metric operates and the specific evaluation dimension it captures. BERTScore F1 [22] operates at the token level and serves as the primary metric for assessing correctness, representing the harmonic mean of precision and recall. BERTScore Recall also operates at the token level, measuring the completeness of the generated interpretation by evaluating how much of the clinically relevant content from the reference definition is captured. BERTScore Precision, likewise operating at the token level, assesses the proportion of generated content that aligns with the reference definition. Cosine similarity, computed using Bio_ClinicalBERT embeddings, operates at the sentence level and captures semantic alignment between the generated interpretation and the reference definition. Finally, the combined score operates at the instance level, calculated as the arithmetic mean of BERTScore F1 and cosine similarity, providing an overall measure of interpretation quality for each instance.

Together, these metrics provide a multi-level evaluation framework capturing both token-level correctness and sentence-level semantic alignment, enabling a more comprehensive assessment of the clinical accuracy of the generated definitions.

### 3.5. Statistical Analysis

Statistical analysis was conducted to summarize the performance of the evaluated models across all test instances. Descriptive statistics were calculated for each evaluation metric, including mean, median, standard deviation, and interquartile range. This provided insight into both the central tendency and variability of model performance. The use of multiple statistical indicators allowed identification of models that demonstrated both high semantic similarity and stable output quality across the dataset. In addition to descriptive statistics, aggregated evaluation scores were computed to facilitate comparison between models. These aggregated measures combined the semantic similarity metrics described in the previous section to obtain an overall representation of model performance for each test instance.

Based on these metrics, models were ranked according to their overall performance. The ranking procedure considered aggregated evaluation scores derived from the combined semantic similarity measures.

### 3.6. Workflow

The overall evaluation pipeline used in this study is illustrated in Figure 1. The workflow began with the extraction and preprocessing of clinical findings from anonymized urological EHR reports. Each extracted finding was treated as an independent input unit and combined with retrieved contextual medical information within the RAG framework. The prepared inputs were then provided to multiple large language models, which generated definitions and interpretations of the identified clinical terms. The generated outputs were stored in a structured dataset together with the original inputs, retrieved references, and ground truth definitions. Finally, the model-generated interpretations were evaluated using automated semantic similarity metrics, including BERTScore and cosine similarity based on ClinicalBERT embeddings. This evaluation pipeline ensured consistent processing across all models and enabled systematic comparison of their interpretive performance.

## 4. Results

This section presents the evaluation results of eight large language models (LLMs) in interpreting urological findings from 40 clinical reports. Model performance was assessed using BERTScore [22] (F1, Precision, Recall) and CBERTScore cosine similarity to measure both lexical and semantic alignment with reference definitions.

### 4.1. Aggregate Performance Across All Models

Before proceeding to a model-by-model comparison, we first examined the aggregate performance across all 320 generated interpretations. Table 3 provides a statistical summary for each evaluation metric across the entire dataset.

The aggregate results indicate generally high performance, with a mean BERTScore F1 of 0.8207 and a mean cosine similarity of 0.9053. However, a closer examination reveals important distributional characteristics. The notable discrepancy between the mean and median for BERTScore F1 (0.8207 vs. 0.8707) points to a negatively skewed distribution. This skewness suggests that while the majority of interpretations achieve high accuracy, a subset of outlier outputs with exceptionally low scores disproportionately lowers the overall mean.

The minimum BERTScore F1 value of 0.0000 is particularly noteworthy, as it confirms that certain models experienced complete failure on specific findings, a critical observation for any potential clinical application. Despite these tail effects, the narrow interquartile ranges (IQR ≈ 0.04 across all metrics) demonstrate that for the central 50% of interpretations, performance is remarkably consistent, with significant instability largely confined to the distribution tails.

Analysis of performance by finding complexity revealed that all models performed better on straightforward findings (mean BERTScore F1 = 0.85) compared to complex findings (mean BERTScore F1 = 0.76). This difference was most pronounced for unstable models, which exhibited catastrophic failures predominantly on complex findings.

As shown in Figure 2, the distribution of BERTScore F1 scores varies considerably across models, revealing distinct performance tiers. The boxplot highlights the differences in median performance, dispersion, and the presence of outliers among the evaluated models.

#### 4.1.1. Top-Tier Performers: Consistent and Clinically Reliable

The top-performing tier comprises DeepSeek-V3.2 [37], GLM-4.6, MiMo-V2-Flash, and Qwen2.5-7B-Instruct. These models exhibit tightly clustered boxplots with medians ranging from 0.879 to 0.889 and narrow interquartile ranges (IQR ≈ 0.020 to 0.034). This consistency across all 40 findings indicates clinically reliable performance. Notably, GLM-4.6 achieves the highest median F1 score (0.8886), while DeepSeek-V3.2 demonstrates the highest upper whisker, suggesting superior performance on a subset of more complex findings.

#### 4.1.2. Mid-Range Models: Reliable but Less Accurate

The mid-range tier consists of Mistral-7B-Instruct-v0.2 [6] and NVIDIA-Nemotron-Nano-9B-v2. Mistral-7B maintains respectable performance with a median of 0.8578 and a tight dispersion (IQR = 0.0227), making it a reliable, if not exceptional, performer. NVIDIA-Nemotron exhibits the narrowest boxplot of all models (IQR = 0.0144), confirming its remarkable consistency. However, this consistency comes at the cost of a lower median value (0.8262), indicating that its interpretations are uniformly adequate but rarely achieve the highest accuracy scores.

#### 4.1.3. Unstable Models: Clinically Unsuitable

The unstable tier includes Kimi-K2-Thinking and gpt-oss-20b, both of which display distributional characteristics that raise serious concerns for clinical use. Kimi-K2 shows an elongated boxplot with a median (0.8783) substantially higher than its mean (0.7928), a classic signature of negative skewness caused by a subset of catastrophic failures. A detailed analysis reveals that on approximately 35 of the 40 findings, this model performs comparably to top-tier systems, but on the remaining five, its performance collapses to below 0.5, generating clinically meaningless output. Furthermore, gpt-oss-20b was even more alarming, with boxplot spans nearly entirely in range from 0 to 1. Its median of 0.86 stands in contrast to its mean of 0.555, which is dragged down by numerous zero-value outliers. This bimodal distribution, where the model either produces highly accurate interpretations or completely fails, renders it clinically unsuitable. Without expert oversight, there is not a reliable mechanism to distinguish successful from failed.

### 4.2. Summary of Model Rankings

Before proceeding to semantic similarity analysis, we first present a consolidated overview of model performance across all four ranking metrics. Table 4 summarizes the rankings of all eight models based on BERTScore F1 mean, BERTScore F1 median, cosine similarity mean, and cosine similarity median. Lower ranks indicate better performance, with 1 being the highest possible rank.

The summary table reveals several notable patterns. DeepSeek-V3.2 demonstrates exceptional performance, securing the top rank in three out of four metrics. GLM-4.6 achieves the highest rank in F1 median but drops to fifth place in both cosine metrics, indicating a literal generation style. Conversely, Mistral-7B shows the opposite pattern, weaker lexical alignment (seventh in F1 median) but strong semantic performance (third in both cosine metrics). The unstable behavior of Kimi-K2-Thinking and gpt-oss-20b is already evident from their inconsistent rankings across metrics.

### 4.3. Semantic Similarity Analysis

To assess whether the generated interpretations capture the underlying meaning of the reference definitions, we evaluated them using CBERTScore cosine similarity. This metric provides insight into semantic alignment beyond mere lexical overlap. Table 5 presents the results, with models sorted by their mean cosine similarity.

While cosine scores broadly correlate with BERTScore F1 rankings (Spearman’s ρ=0.86), several notable dissociations emerge. DeepSeek-V3.2 maintains its leadership with a mean cosine of 0.9517, confirming that its high lexical accuracy reflects genuine semantic alignment. More interestingly, Mistral-7B achieves the third-highest cosine similarity (0.9490) despite ranking fifth by BERTScore F1. This indicates that its interpretations, while less lexically precise, successfully capture the semantic essence of the reference definitions.

Conversely, GLM-4.6 drops from second place in F1 to fifth place in cosine similarity (0.9428). This suggests a more literal, less paraphrased generation style that maintains terminological precision but may not restructure information as flexibly. This trade-off between lexical precision and semantic flexibility has important implications for patient-facing applications, where paraphrasing may enhance comprehensibility for non-expert audiences.

The high standard deviations observed for Kimi-K2-Thinking and gpt-oss-20b further corroborate the bimodal distribution patterns identified in the BERTScore analysis, reinforcing their classification as unstable models.

### 4.4. Individual Model Ranking Analysis

To provide a more granular view of model performance, Figure 3, Figure 4, Figure 5, Figure 6, Figure 7, Figure 8, Figure 9 and Figure 10 present individual ranking profiles for each evaluated model across four metrics: BERTScore F1 mean, BERTScore F1 median, cosine similarity mean, and cosine similarity median. Lower ranks indicate better performance, with 1 being the highest possible rank.

#### 4.4.1. DeepSeek-V3.2 Ranking Analysis

Figure 3 illustrates the ranking of DeepSeek-V3.2 across four evaluation metrics. The model demonstrates exceptional performance, achieving the highest possible rank (1) in three out of four metrics: F1 mean, cosine mean, and cosine median. Its only relative weakness appears in F1 median, where it ranks third. This pattern suggests that while DeepSeek-V3.2 produces consistently excellent interpretations, a small subset of outputs may deviate slightly from the reference definitions in lexical terms, though semantic alignment remains flawless. Overall, the model establishes itself as the most balanced and clinically reliable performer in the evaluation.

**Figure 3:**
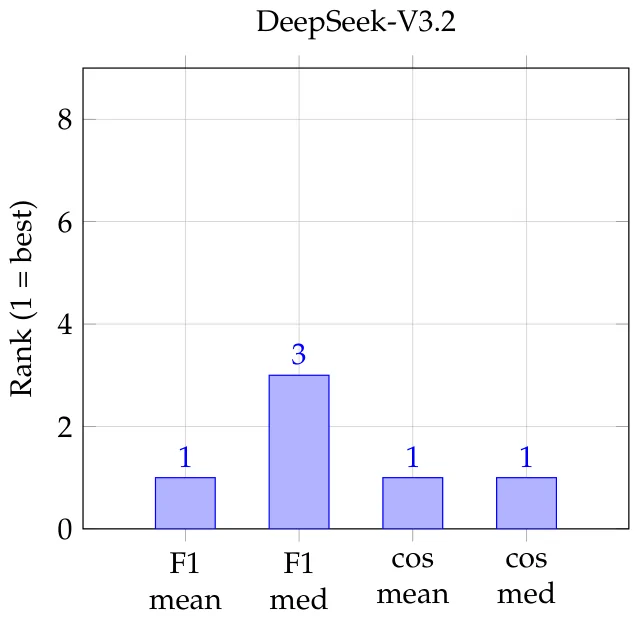
DeepSeek-V3.2 Ranking Across Metrics (F1: F1-Score; cos: cosine similarity; med: median).

#### 4.4.2. MiMo-V2-Flash Ranking Analysis

Figure 4 presents the ranking profile of MiMo-V2-Flash. The model exhibits remarkable consistency across all four metrics, securing second place in F1 median, cosine mean, and cosine median, while placing third in F1 mean. This uniformity indicates that MiMo-V2-Flash generates interpretations of stable quality without significant fluctuations between lexical accuracy and semantic alignment. Its performance places it firmly in the top tier, just behind DeepSeek-V3.2, making it a reliable candidate for clinical applications where consistency is paramount.

**Figure 4:**
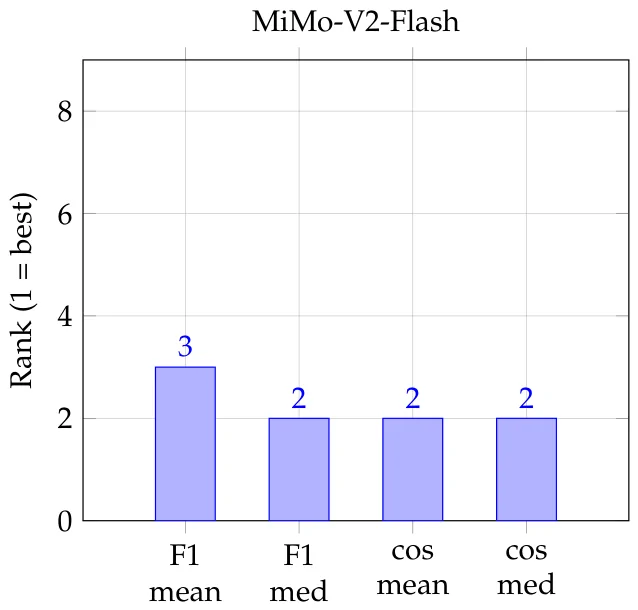
MiMo-V2-Flash Ranking Across Metrics (F1: F1-Score; cos: cosine similarity; med: median).

#### 4.4.3. GLM-4.6 Ranking Analysis

Figure 5 reveals a distinctive performance pattern for GLM-4.6. The model achieves the highest rank in F1 median (1) and second place in F1 mean (2), demonstrating exceptional lexical precision. However, its semantic alignment metrics tell a different story, ranking fifth in both cosine mean and cosine median. This dissociation confirms that GLM-4.6 employs a more literal generation strategy, preserving exact terminology at the cost of paraphrastic flexibility. While this approach yields terminologically precise outputs, it may limit the model’s effectiveness in patient-facing contexts where rephrasing enhances comprehension.

#### 4.4.4. Qwen2.5-7B-Instruct Ranking Analysis

Figure 6 showcases the performance of Qwen2.5-7B-Instruct, which achieves the same rank (4) across all four evaluation metrics. This perfect uniformity is itself a notable finding; the model neither excels nor underperforms in any specific dimension, but rather delivers consistently average results. While Qwen does not reach the heights of top-tier models, its balanced profile ensures predictable performance without unexpected failures, positioning it as a dependable mid-range option.

**Figure 5:**
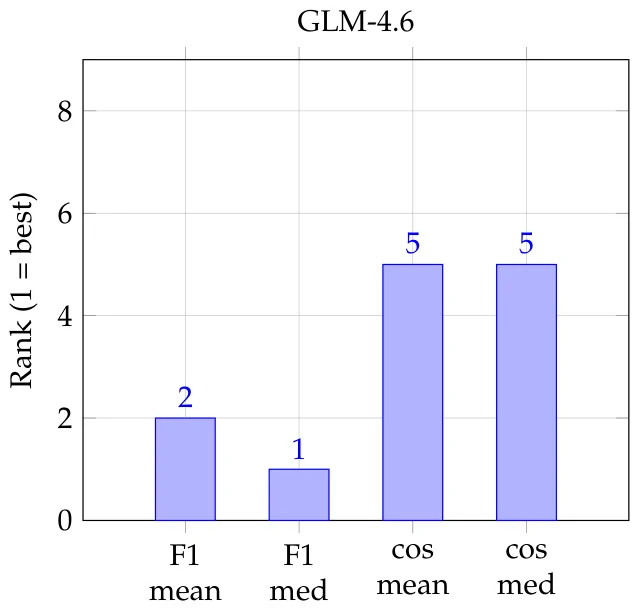
GLM-4.6 Ranking Across Metrics (F1: F1-Score; cos: cosine similarity; med: median).

**Figure 6:**
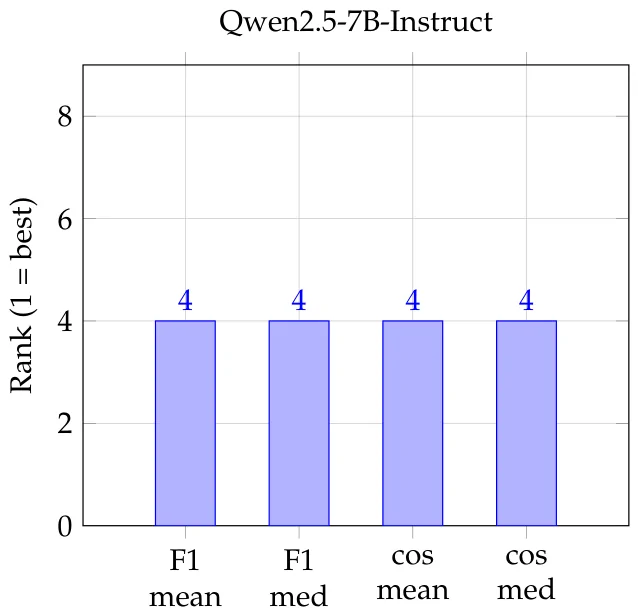
Qwen2.5-7B-Instruct Ranking Across Metrics (F1: F1-Score; cos: cosine similarity; med: median).

#### 4.4.5. Mistral-7B-Instruct-v0.2 Ranking Analysis

Figure 7 illustrates the inverse pattern to GLM-4.6. Mistral-7B ranks fifth in F1 mean and seventh in F1 median, indicating relatively weaker lexical alignment, but achieves an impressive third place in both cosine mean and cosine median. This profile suggests that Mistral-7B excels at capturing the semantic essence of clinical findings even when its word-for-word match with reference definitions is less precise. For applications prioritizing meaning over exact terminology, such as patient education or clinical communication, this semantic flexibility represents a significant strength.

#### 4.4.6. Kimi-K2-Thinking Ranking Analysis

Figure 8 exposes the unstable behavior of Kimi-K2-Thinking. The model ranks seventh in F1 mean, fifth in F1 median, and sixth in both cosine metrics. The discrepancy between mean and median, particularly the two-point gap in F1 rankings, signals the presence of catastrophic outliers that drag down average performance despite acceptable median scores. This pattern confirms that Kimi-K2-Thinking performs well on most findings but collapses completely on a subset, rendering it clinically unsuitable due to the unpredictability of its failures.

**Figure 7:**
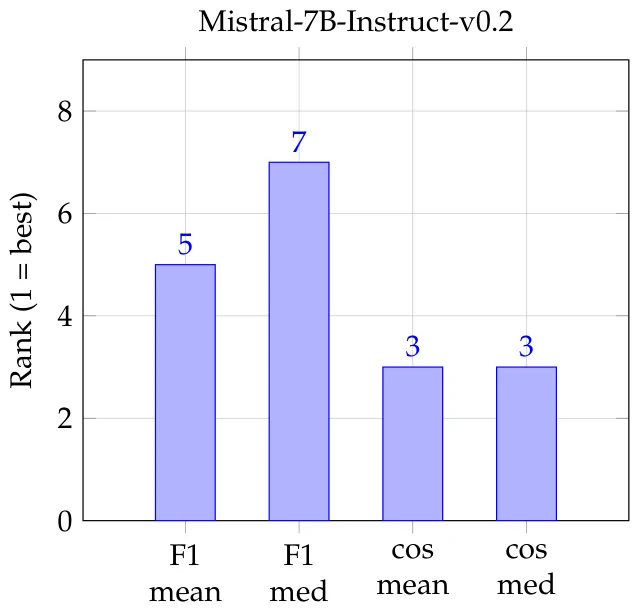
Mistral-7B-Instruct-v0.2 Ranking Across Metrics (F1: F1-Score; cos: cosine similarity; med: median).

**Figure 8:**
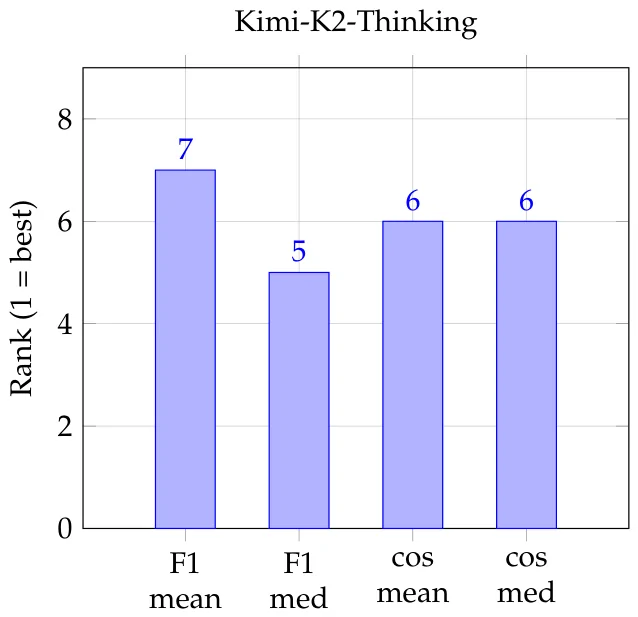
Kimi-K2-Thinking Ranking Across Metrics (F1: F1-Score; cos: cosine similarity; med: median).

#### 4.4.7. NVIDIA-Nemotron-Nano-9B-v2 Ranking Analysis

Figure 9 presents the ranking profile of NVIDIA-Nemotron-Nano-9B-v2. The model consistently occupies the lower ranks, sixth in F1 mean, eighth in F1 median, seventh in cosine mean, and eighth in cosine median. While these numbers appear unimpressive, they reflect a specific design trade-off: NVIDIA-Nemotron prioritizes consistency over peak performance. Earlier analysis confirmed that this model exhibits the narrowest interquartile range of all tested systems, meaning its outputs are uniformly adequate but rarely excellent. In clinical contexts where predictability matters more than occasional brilliance, this stability may still hold value.

**Figure 9:**
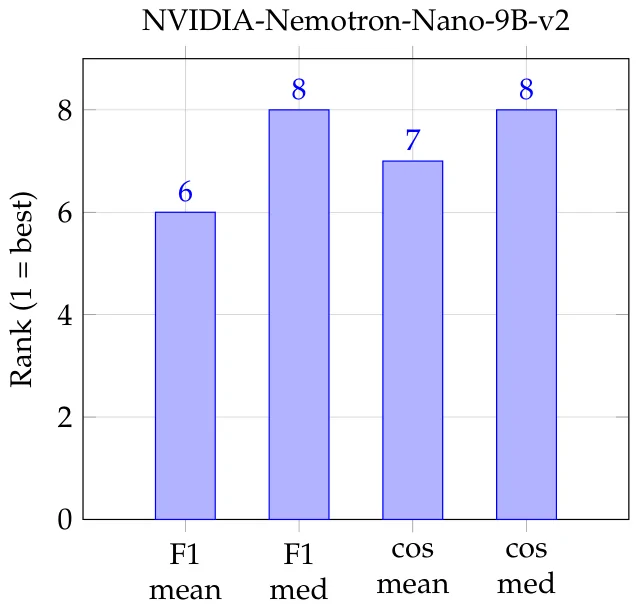
NVIDIA-Nemotron-Nano-9B-v2 Ranking Across Metrics (F1: F1-Score; cos: cosine similarity; med: median).

#### 4.4.8. gpt-oss-20b Ranking Analysis

Figure 10 reveals the most concerning performance profile in the evaluation. gpt-oss-20b ranks last (8) in F1 mean and cosine mean, sixth in F1 median, and seventh in cosine median. The dramatic gap between its median and mean ranking, particularly the two-point difference in F1, confirms the bimodal distribution identified earlier: the model either produces highly accurate interpretations or completely fails, with little in between. This catastrophic instability, combined with its bottom-tier rankings, eliminates any possibility of clinical deployment. Without expert oversight to separate successes from failures, gpt-oss-20b poses unacceptable risks in medical contexts.

**Figure 10:**
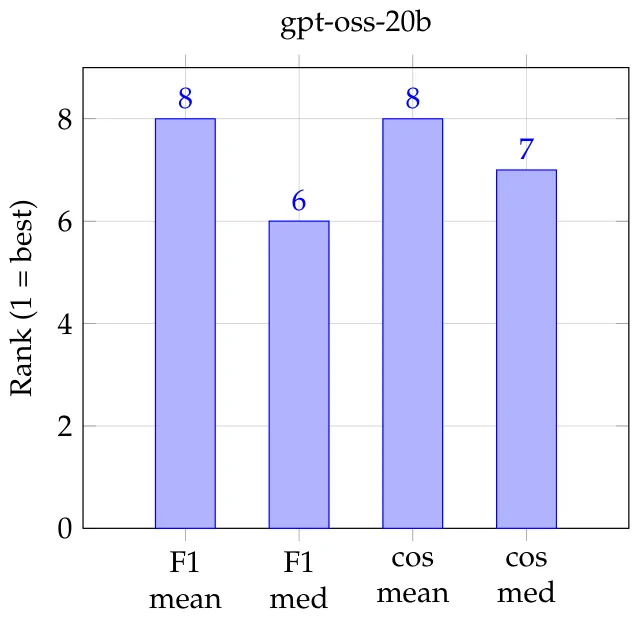
gpt-oss-20b Ranking Across Metrics (F1: F1-Score; cos: cosine similarity; med: median).

### 4.5. Qualitative Analysis: Error Patterns and Exemplar Cases

While quantitative metrics provide a robust numerical framework, they do not fully capture the nature of the generated interpretations. A qualitative examination of raw model outputs reveals distinct error patterns that metrics alone cannot illuminate.

- Top-performing models consistently: (a) correctly expand abbreviations using context, (b) connect related findings (e.g., linking PSA to prostate volume), (c) provide balanced explanations that neither overstate nor understate clinical significance, and (d) avoid unsupported diagnostic speculation.
- Mid-range models typically: (a) correctly interpret individual findings but miss interrelationships, (b) provide accurate but incomplete explanations (omitting key contextual information), and (c) occasionally introduce minor inaccuracies that do not fundamentally distort clinical meaning.
- Unstable models exhibit catastrophic failure patterns including: (a) complete hallucination (inventing non-existent findings or recommendations), (b) contextual disregard (ignoring clinically relevant numerical values), (c) diagnostic overreach (confidently asserting unsupported diagnoses), and (d) terminological invention (creating plausible-sounding but medically meaningless terminology).

#### 4.5.1. *Case Study 1: Successful Interpretation by DeepSeek-V3.2*

The following example illustrates a clinically accurate and well-balanced interpretation generated by a top-performing model:

- Original finding: “Ultrasound: Left kidney normal size, parenchyma thinned, DE 4 mm.”
- Reference definition: “DE (echoparenchymal differentiation) indicates the clarity of distinguishing renal parenchyma from medulla. DE 4 mm is within normal limits.”
- Model interpretation: “The finding describes a left kidney of normal size with parenchymal thinning. DE (echoparenchymal differentiation) is 4 mm, which is within normal limits. Parenchymal thinning may be a consequence of aging or chronic changes, but without significant impact on function given the normal DE value.”
- Analysis: The model correctly interprets the abbreviation DE, connects it with the parenchymal thinning finding, and provides a balanced explanation that is neither overly alarming nor overly optimistic. The interpretation is complete, accurate, and appropriate for patient communication.

#### 4.5.2. *Case Study 2: Severe Hallucination by gpt-oss-20b*

In contrast, the following example demonstrates a severe hallucination, highlighting the potential risks of unreliable model behavior:

- Original finding: “PSA 4.2 ng/mL, normal digital rectal examination, prostate ultrasound: volume 48 mL.”
- Reference definition: “PSA above 4.0 ng/mL is considered elevated. Prostate volume of 48 mL indicates benign hyperplasia (BPH), which is a common cause of mildly elevated PSA.”
- Model interpretation (hallucination): “PSA 4.2 is significantly elevated and almost certainly indicates prostate cancer. Urgent biopsy and MPV examination are needed. Prostate volume of 48 mL is normal.”
- Analysis: The model (1) incorrectly interprets prostate volume as normal, (2) ignores the more likely diagnosis (BPH), (3) adds unsupported recommendations for biopsy, and (4) creates completely unnecessary alarm. This interpretation is clinically dangerous and illustrates why models with bimodal failure patterns are unsuitable for medical applications.

### 4.6. Correlation Analysis of Evaluation Metrics

To assess the consistency and redundancy of the four evaluation metrics, we computed Spearman rank correlation coefficients between them. Table 6 presents the correlation matrix, revealing how closely related the different measurement approaches are.

The correlation analysis reveals several important patterns. The strong correlation between cosine mean and cosine median (0.98) indicates that semantic similarity measurements are highly stable regardless of whether mean or median is used. Similarly, F1 mean and F1 median show a strong correlation (0.94), confirming their consistency. The moderate correlation between lexical (F1-based) and semantic (cosine-based) metrics (ρ≈0.82 to 0.86) suggests that while related, they capture distinct aspects of interpretation quality, justifying their combined use in this evaluation. Notably, the unstable models (Kimi-K2, gpt-oss-20b) contribute to the observed variance between metric families.

### 4.7. Summary of Key Findings

The synthesis of quantitative and qualitative analyses yields five principal conclusions:

1. Clear performance stratification: Models separate into three distinct tiers, top-performing (DeepSeek, GLM, MiMo, Qwen), mid-range (Mistral, NVIDIA), and clinically unacceptable (Kimi, gpt-oss), based on accuracy, consistency, and stability.
2. Median as a reliability indicator: The divergence between mean and median effectively identifies unstable models. Medians provide more clinically meaningful estimates of typical performance than means, which are disproportionately affected by catastrophic outliers.
3. Lexical vs. semantic trade-offs: Models exhibit different generation strategies. Some prioritize terminological precision (GLM-4.6), while others demonstrate greater semantic flexibility (Mistral-7B). This distinction has important implications for different use cases, particularly patient-facing applications where comprehensibility is paramount.
4. Stability as a clinical prerequisite: Low standard deviation and narrow interquartile range, exemplified by NVIDIA-Nemotron, may be preferable to higher mean accuracy accompanied by occasional catastrophic failure. Predictability is essential in clinical contexts, where even rare errors can have serious consequences.
5. Architecture over geography: While four of the five top-performing models originate from China, performance differences more likely reflect model architecture, training data, and optimization rather than geographic origin per se.

## 5. Discussion

LLMs are increasingly being explored as tools for supporting the interpretation of clinical documentation, but medical reports frequently contain specialized terminology that can be difficult to interpret without domain expertise. This study investigated whether LLMs can generate concise and clinically meaningful definitions of urological findings extracted from authentic electronic health record documentation. The results suggest that LLMs are capable of producing coherent explanations that often show strong semantic similarity with reference definitions, consistent with findings from other medical domains [20,21,23].

Furthermore, one of the main strengths observed is the ability of LLMs to transform short fragments of clinical text into structured and linguistically clear medical definitions. In many cases, the generated outputs captured the essential meaning of the clinical finding and aligned well with reference interpretations according to automated similarity metrics such as BERTScore [22]. This indicates that LLMs may be useful for supporting the explanation of specialized terminology in clinical documentation.

However, several challenges remain. Although the prompting strategy constrained the models to produce short definitions without additional commentary, occasional cases of hallucination were observed, where models introduced information not explicitly present in the source text. Research on hallucination mitigation [11,12] has identified similar patterns across various LLM applications. Such inaccuracies represent a significant concern in medical contexts, where even minor deviations from factual information may lead to misinterpretation. In addition, model performance varied across systems, suggesting that architecture, training data, and prompt sensitivity can influence the reliability of generated interpretations. The performance of models like LLaMA 3 [5] and Mistral 7B [6] in our evaluation aligns with their general capabilities reported in the literature.

From a clinical perspective, these findings suggest that LLMs may be best suited as decision-support tools rather than autonomous interpreters of medical documentation. Their ability to generate explanations could assist clinicians, researchers, or students in understanding unfamiliar terminology, but human oversight remains essential to ensure clinical accuracy and safety. This conclusion aligns with recommendations from recent reviews of LLM applications in urology [7,8] and broader discussions of trustworthy AI in healthcare [16].

It is important to emphasize that the automated semantic similarity metrics used in this study measure agreement with reference definitions, not clinical reliability per se. Establishing clinical reliability would require prospective validation studies with human expert assessment in real clinical workflows. The findings presented here should therefore be interpreted as indicators of agreement rather than direct evidence of clinical safety or effectiveness.

Furthermore, the deployment of LLMs in clinical settings raises important ethical considerations beyond technical accuracy, including patient privacy and data governance, algorithmic fairness, transparency, accountability, and regulatory compliance [2,3]. These dimensions must be addressed alongside performance optimization to ensure responsible and equitable implementation of AI tools in healthcare.

Moreover, the limitations of this study should be acknowledged. Firstly, the evaluation was conducted on a relatively small dataset consisting of 40 urological reports from a single clinical center (Clinical Hospital Center Rijeka), which limits the generalizability of the findings. What is more, the evaluation relied primarily on text-based similarity metrics, and while these measures provide useful quantitative indicators of semantic similarity, they may not fully capture the clinical nuance that human experts would recognize. Additionally, the identification of hallucinations was based on predefined evaluation criteria, which may lead to partial under- or overestimation of model errors.

The dataset, while diverse in terms of urological subdomains and complexity levels, remains limited in size. Future studies should include larger and more diverse datasets to validate the findings across broader patient populations and clinical settings.

Future work could therefore include larger and more diverse datasets, evaluations across multiple medical specialties, and human expert validation. In addition, integrating approaches such as RAG [17], which link model outputs to authoritative medical sources, may further improve the reliability and transparency of LLM-based clinical interpretation systems. Building on benchmarks like GLUE [19] and ClinicBench [20], developing urology-specific evaluation frameworks would also be valuable. Finally, exploring the integration of such systems within EHR environments could help determine their practical utility in real-world clinical workflows.

## 6. Conclusions

This study evaluated the ability of LLMs to generate concise and clinically meaningful definitions of urological findings extracted from electronic health record documentation. Using automated semantic evaluation metrics including BERTScore, the results demonstrate that LLMs can produce interpretations that show substantial semantic alignment with reference definitions derived from the clinical literature. Specifically, the highest-performing models achieved a mean BERTScore F1 of 0.927 and a mean cosine similarity of 0.952, while the overall mean across all models was 0.821 for BERTScore F1 and 0.905 for cosine similarity, indicating strong lexical and semantic consistency with medically verified definitions despite variability in model performance. These findings suggest that modern language models such as DeepSeek-V3.2 and Mistral-7B possess considerable potential for supporting the interpretation and explanation of specialized medical terminology.

At the same time, this study highlights the importance of clinically informed evaluation frameworks when assessing LLM-generated outputs in medical contexts. While automated semantic similarity metrics provide useful quantitative indicators of alignment with reference definitions, they should be complemented with clinically aware validation procedures to ensure that generated explanations remain accurate, relevant, and safe for potential clinical use. The variability observed across models underscores the need for domain-specific evaluation rather than reliance on general-purpose benchmarks alone.

Overall, the results indicate that LLMs may serve as supportive tools for explaining clinical findings, particularly in contexts where rapid interpretation of specialized terminology is required. However, due to the potential for inaccuracies or hallucinated information, such systems should currently be considered decision-support mechanisms rather than autonomous clinical interpreters. This cautious approach is consistent with recommendations from the broader literature on LLM applications in healthcare.

Future research could focus on expanding the evaluation to larger and more diverse clinical datasets and extending the analysis across multiple medical specialties to better assess generalizability. In addition, integrating RAG approaches that link model outputs to authoritative medical sources may improve reliability and transparency. Finally, exploring the integration of such systems within EHR environments could help determine their practical utility in real-world clinical workflows.

## Figures and Tables

**Figure 1:**
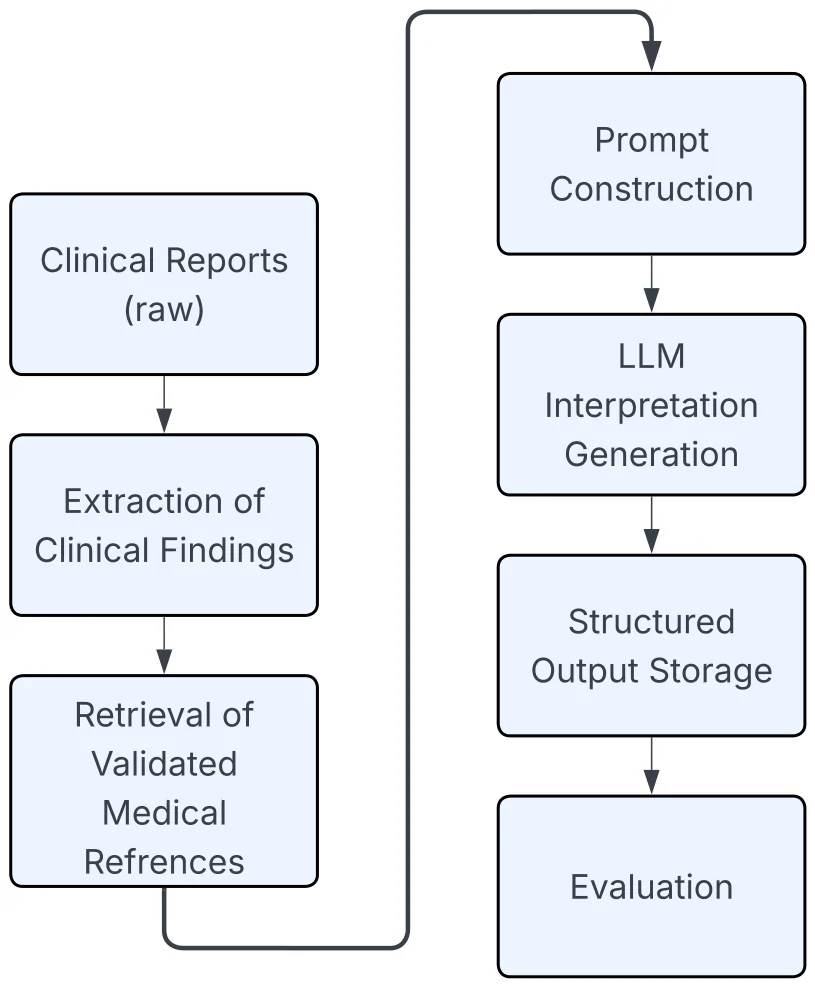
Workflow of the evaluation pipeline for LLM interpretation of urological EHR findings.

**Figure 2:**
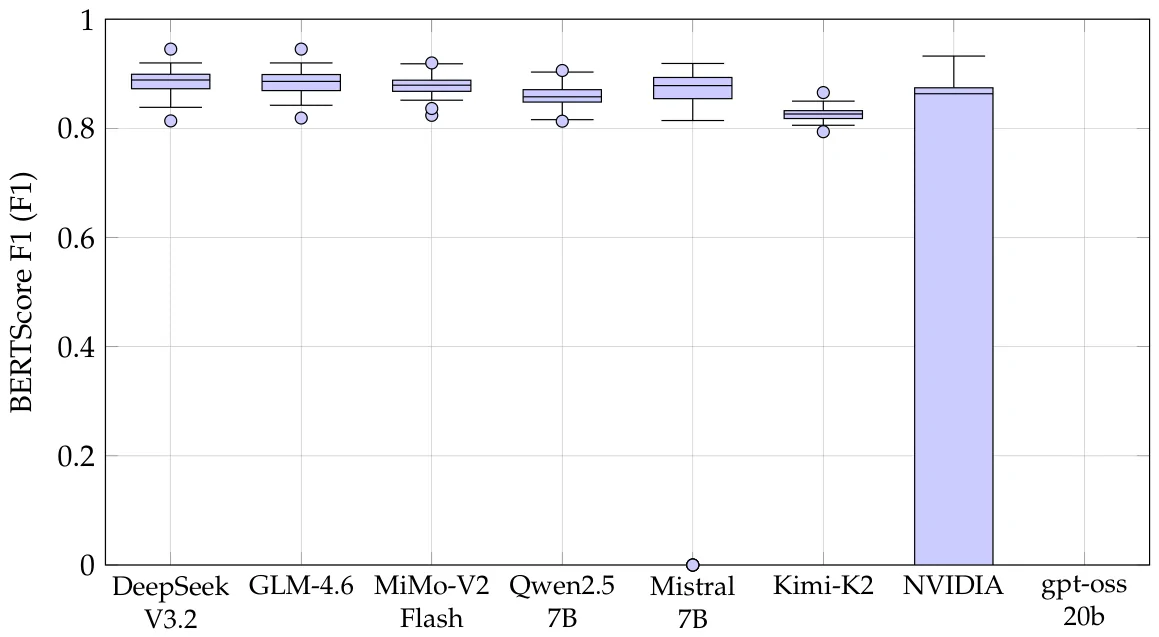
Model Comparison by BERTScore F1-Measure (F1: F1-Score).

**Table 1:** Prompt Template Used for LLM Interpretation Generation.

Component	Content
System Instruction	“You are a medical expert specialized in urology. Your task is to provide a concise, clinically accurate definition of the given urological term or finding based exclusively on the provided text segment and retrieved context. Do not include additional explanations, lists, or bullet points.”
User Instruction	“Based on the following clinical finding and retrieved context, provide a clear and concise medical definition of the term or finding. Preserve the medical terminology present in the input text and do not translate it. Clinical Finding: [extracted text segment]. Retrieved Context: [curated reference material].”

**Table 2:** Summary of Automated Evaluation Metrics (BERT: Bidirectional Encoder Representations from Transformers; F1: F1-Score).

Metric	Level	Dimension
BERTScore F1	Token	Correctness
BERTScore Recall	Token	Completeness
BERTScore Precision	Token	Completeness
Cosine Similarity (Clinical_BERT)	Sentence	Alignment
Combined Score	Instance	Overall

**Table 3:** Overall descriptive statistics for all models (N = 320) (F1: F1-Score; CBERT: Clinical BERT; IQR: Interquartile Range).

Metric	Mean	Median	IQR	Min	Max
BERTScore F1	0.8207	0.8707	0.0428	0.0000	0.9452
cosine CBERT	0.9053	0.9460	0.0429	0.3260	0.9870
Combined	0.8630	0.9099	0.0406	0.1630	0.9661

**Table 4:** Summary of Model Rankings Across All Metrics (F1: F1-Score; cos: cosine similarity; med: median).

Model	F1 Mean	F1 med	cos Mean	cos med
DeepSeek-V3.2	1	3	1	1
MiMo-V2-Flash	3	2	2	2
GLM-4.6	2	1	5	5
Qwen2.5-7B-Instruct	4	4	4	4
Mistral-7B-Instruct-v0.2	5	7	3	3
Kimi-K2-Thinking	7	5	6	6
NVIDIA-Nemotron-Nano-9B-v2	6	8	7	8
gpt-oss-20b	8	6	8	7

**Table 5:** Semantic Similarity Results (CBERTScore Cosine) (CBERT: Clinical BERT; SD: Standard Deviation; IQR: Interquartile Range).

Model	Mean	SD	Median	IQR	Rank
DeepSeek-V3.2	0.9517	0.0231	0.9549	0.0262	1
MiMo-V2-Flash	0.9486	0.0236	0.9546	0.0246	2
Mistral-7B	0.9490	0.0216	0.9531	0.0286	3
Qwen2.5-7B	0.9471	0.0207	0.9509	0.0259	4
GLM-4.6	0.9428	0.0255	0.9499	0.0317	5
Kimi-K2	0.8883	0.1791	0.9489	0.0403	6
NVIDIA	0.8928	0.0231	0.8996	0.0263	7
gpt-oss-20b	0.7221	0.2855	0.9201	0.5929	8

**Table 6:** Spearman Correlation Matrix Between Evaluation Metrics (F1: F1-Score; cos: cosine similarity; med: median).

Metric	F1 Mean	F1 med	cos Mean	cos med
F1 mean	1.00	0.94	0.86	0.85
F1 med	0.94	1.00	0.82	0.81
cos mean	0.86	0.82	1.00	0.98
cos med	0.85	0.81	0.98	1.00

## Data Availability

The dataset used in this study consists of anonymized real EHR reports collected from routine clinical practice at the Clinical Hospital Center Rijeka. Due to patient privacy and ethical restrictions, the data cannot be publicly shared. The data are available from the corresponding author upon reasonable request and with appropriate institutional approval.

## Abbreviations

The following abbreviations are used in this manuscript:

AI	Artificial Intelligence
BERT	Bidirectional Encoder Representations from Transformers
BPH	Benign Prostatic Hyperplasia
DE	Echoparenchymal Differentiation
EHR	Electronic Health Record
F1	F1-Score
GLUE	General Language Understanding Evaluation
IQR	Interquartile Range
LLM	Large Language Model
PSA	Prostate-Specific Antigen
RAG	Retrieval-Augmented Generation
SD	Standard Deviation
